# Azithromycin and Ciprofloxacin Resistance in *Salmonella* Bloodstream Infections in Cambodian Adults

**DOI:** 10.1371/journal.pntd.0001933

**Published:** 2012-12-13

**Authors:** Erika R. Vlieghe, Thong Phe, Birgit De Smet, Chhun H. Veng, Chun Kham, Sophie Bertrand, Raymond Vanhoof, Lut Lynen, Willy E. Peetermans, Jan A. Jacobs

**Affiliations:** 1 Department of Clinical Sciences, Institute of Tropical Medicine, Antwerp (ITM), Antwerp, Belgium; 2 Sihanouk Hospital Centre of Hope (SHCH), Phnom Penh, Cambodia; 3 Belgian Reference Center for Salmonella, Scientific Institute of Public Health (IPH), Brussels, Belgium; 4 Department of Internal Medicine, University Hospital, Leuven, Belgium; Massachusetts General Hospital, United States of America

## Abstract

**Background:**

*Salmonella enterica* is a frequent cause of bloodstream infection (BSI) in Asia but few data are available from Cambodia. We describe *Salmonella* BSI isolates recovered from patients presenting at Sihanouk Hospital Centre of Hope, Phnom Penh, Cambodia (July 2007–December 2010).

**Methodology:**

Blood was cultured as part of a microbiological prospective surveillance study. Identification of *Salmonella* isolates was performed by conventional methods and serotyping. Antibiotic susceptibilities were assessed using disk diffusion, MicroScan and E-test macromethod. Clonal relationships were assessed by Pulsed Field Gel Electrophoresis; PCR and sequencing for detection of mutations in Gyrase and Topoisomerase IV and presence of *qnr* genes.

**Principal Findings:**

Seventy-two *Salmonella* isolates grew from 58 patients (mean age 34.2 years, range 8–71). Twenty isolates were identified as *Salmonella* Typhi, 2 as *Salmonella* Paratyphi A, 37 as *Salmonella* Choleraesuis and 13 as other non-typhoid *Salmonella* spp. Infection with human immunodeficiency virus (HIV) was present in 21 of 24 (87.5%) patients with *S.* Choleraesuis BSI. Five patients (8.7%) had at least one recurrent infection, all with *S.* Choleraesuis; five patients died. Overall, multi drug resistance (i.e., co-resistance to ampicillin, sulphamethoxazole-trimethoprim and chloramphenicol) was high (42/59 isolates, 71.2%). *S.* Typhi displayed high rates of decreased ciprofloxacin susceptibility (18/20 isolates, 90.0%), while azithromycin resistance was very common in *S.* Choleraesuis (17/24 isolates, 70.8%). Two *S.* Choleraesuis isolates were extended spectrum beta-lactamase producer.

**Conclusions and Significance:**

Resistance rates in *Salmonella* spp. in Cambodia are alarming, in particular for azithromycin and ciprofloxacin. This warrants nationwide surveillance and revision of treatment guidelines.

## Author Summary

*Salmonella enterica* is a bacterium that causes important morbidity and mortality worldwide, especially in tropical low-resource settings. Over the past two decades, increasing rates of resistance for the commonly available oral antibiotics have been reported in *Salmonella* spp., especially from South(east) Asia. As microbiology laboratories are extremely scarce in Cambodia, data on the presence and resistance of *Salmonella* spp. in this country are limited. The authors describe the different types and antibiotic resistance of 72 *Salmonella* isolates from blood cultures sampled in 58 adult Cambodian patients with fever. The most common serovars were *Salmonella* Typhi and *Salmonella* Choleraesuis. The latter serovar causes illness in pigs, and may occasionally infect humans through contact with contaminated animals or environments, especially those with decreased immunity. The authors noted resistance for the first line oral antibiotics in nearly three quarters of all *Salmonella* isolates. In addition, 90% of all *S.* Typhi had decreased susceptibility for ciprofloxacin, while around 70% of *S.* Choleraesuis showed resistance to azithromycin. These results seriously limit the treatment options for typhoid fever and other invasive Salmonella infections and warrant nationwide surveillance of antibiotic resistance. This is the first report to describe such high rates of azithromycin resistance in *Salmonella enterica*.

## Introduction

*Salmonella enterica* is an important cause of morbidity and mortality worldwide [1], [2]. *Salmonella enterica* serovar Typhi is the etiologic agent of typhoid fever while non-typhoid *Salmonella* spp. (NTS) are associated with gastroenteritis and invasive infections in children, the elderly and immune compromised [3]. Both *S.* Typhi and NTS are among the most frequent pathogens causing bloodstream infections (BSI) in tropical low-resource settings [4]. The highest incidence of *Salmonella* infections worldwide occurs in Asia [1], [5], mainly in South and Southeast Asia, where isolates show high rates of antibiotic resistance [6], [7]. Although fluoroquinolones are drugs of choice to treat invasive *Salmonella* infections, decreased susceptibility to ciprofloxacin (DCS) is increasing quickly worldwide [2]. Azithromycin and ceftriaxone have been recommended as treatment alternatives for typhoid fever in case of DCS [8]–[10].

Little is known about the epidemiology and the extent of antibiotic resistance in invasive human *Salmonella* infections in Cambodia.

As part of a microbiological surveillance study on the causes of BSI in Cambodian adults and an antibiotic stewardship program, we aimed to assess the antibiotic resistance patterns of invasive salmonellosis in this population.

## Methods

### Study setting and period

Sihanouk Hospital Centre of HOPE (SHCH) is a 40-bed non-government referral hospital in Phnom Penh, Cambodia. Microbiological services were installed in 2005. From July 2007 until June 2011 a prospective BSI surveillance program was carried out.

### Patients and blood culture sampling

From all adult patients presenting with signs of the Systemic Inflammatory Response Syndrome (SIRS) [11], venous blood (2×10 ml) was drawn for culture with registration of demographic and clinical data. Patients were identified with a unique hospital number. Blood was cultured in home-made Brain Heart Infusion broth bottles (BIO-RAD, Berkeley, California) (July 2007–March 2009) and from April 2009 onward in BacT/ALERT culture bottles (bioMérieux, Marcy l'Etoile, France). Blood cultures were incubated for 7 days at 35°C and daily monitored for growth by visual inspection of the broth or the chromogenic growth indicator respectively. As part of standard patient care, isolates were identified by conventional biochemical tests and assessed for antibiotic susceptibility by disk diffusion. Isolates were stored at −70°C on porous beads in cryopreservative (Microbank, Pro-Lab Diagnostics, Richmond Hill, Canada).

### Microbiological work-up of isolates

Isolates identified as *Salmonella* spp. at SHCH were retrieved from −70°C, checked for purity and further worked up at the Institute of Tropical Medicine (Antwerp, Belgium) and the Scientific Institute of Public Health (Brussels, Belgium). Serotyping was carried out by slide agglutination with commercial antisera according to the Kauffmann-White scheme [12].

Clonal relationships were assessed by pulsed field gel electrophoresis (PFGE) according to the PulseNet Europe protocol [13]. Genomic DNA was digested with XbaI restriction enzymes (New-England Biolab, Leusden, Netherlands), *S.* Braenderup H9812 was used as a size marker. Profiles were analyzed using the Dice coefficient [14] and the unweighted-pair group method using average linkages, with a tolerance of 1%.

For the compilation of the resistance data, only the first isolate per BSI episode (defined as a 14-day period following the first day of BSI diagnosis) was considered. Recurrent infections were defined as a new BSI episode with an identical *Salmonella* serovar at least 14 days after the former isolate and after appropriate treatment of the patient. Recurrent isolates were considered as duplicate isolates and not compiled into the resistance overview; their resistance data were considered separately.

Antibiotic susceptibilities were assessed by disk diffusion (using Neo-Sensitabs™, Rosco Diagnostica, Taastrup, Denmark) and MicroScan (Combo 42, Siemens Healthcare Diagnostics, Deerfield, USA). Minimal inhibitory concentrations (MIC) for nalidixic acid (NA), ciprofloxacin, chloramphenicol and azithromycin were determined using the E-test macromethod (bioMérieux).

Breakpoints were those defined by the Clinical Laboratory Standards Institute [15]; intermediately resistant isolates were considered as resistant. DCS was defined according to European Committee on Antimicrobial Susceptibility testing (EUCAST) guidelines, *i.e.* a MIC-value for ciprofloxacin >0.064 µg/mL [16]. Multidrug resistance (MDR) was defined as co-resistance to the first line antibiotics ampicillin, chloramphenicol and sulphamethoxazole-trimethoprim (SMX-TMP). For azithromycin and *Enterobacteriaceae*, no breakpoints have been published. EUCAST mentions treatment of *S.* Typhi infections with a MIC≤16 µg/mL and a recent publication proposed 16 µg/ml as ‘epidemiological cutoff’ value for wild type *Salmonella* spp. [17]. Detection and identification of ESBL producing *bla* genes was performed by a commercial multiplex ligation PCR microarray CT 101 (Check-Points Health BV, Wageningen, The Netherlands) [18].

Screening for mutations in the quinolone resistance-determining region (QRDR) was performed by amplification of a fragment of the *gyrA*, *gyrB*, and *parC* genes containing the QRDR as previously described [19] and sequencing of the fragments on a CEQ 2000 DNA sequencer (Beckman Coulter, High Wycombe, United Kingdom), using the DTSC-2 method. The sequences were compared and analyzed by Genestream software (Institut de Génétique Humaine, Montpellier, France). The presence of the plasmid-mediated quinolone resistance *qnr* genes (*qnrA*, *qnrB*, and *qnrS*) was determined using PCR [20]

### Statistical analysis

Data were entered in Access and Excel databases (Microsoft Corporation, Redmond, Washington, USA). Risk factors were assessed by univariate analysis using the Χ^2^ with STATA software (Statacorp, College Station, Texas). Differences were considered statistically significant at p-values<0.05.

## Results

### Demographic and clinical data

**Figure 1:**
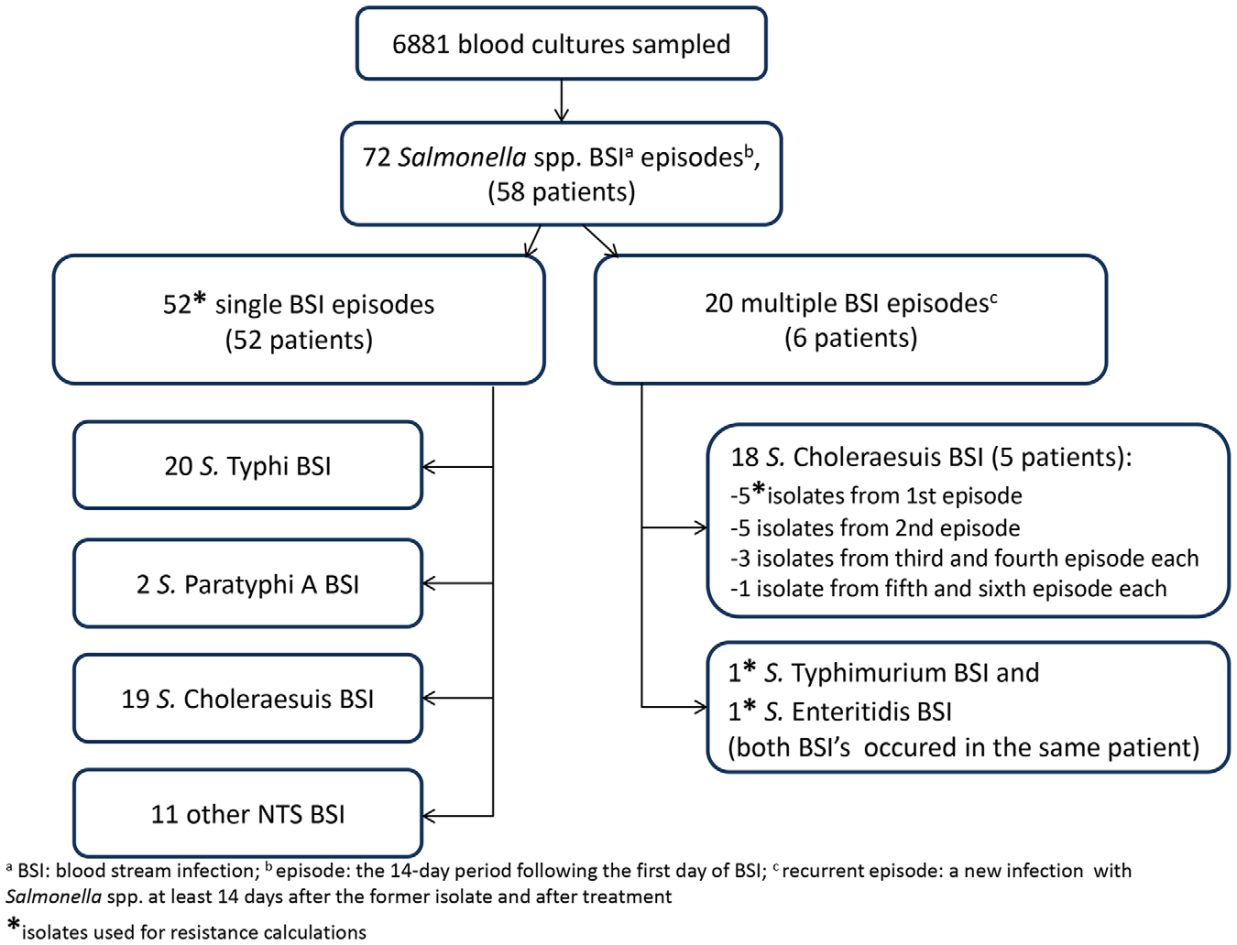
Flow chart of blood stream infection (BSI) episodes, patients and corresponding serovars.

From 6881 blood cultures drawn during the study period, 72 non-duplicate *Salmonella enterica* isolates were recovered from 58 adult patients, representing 11.5% of all clinically significant organisms (CSO). These isolates were recovered from 59 first BSI episodes and 13 recurrent episodes (Figure 1). The serovars included *S.* Choleraesuis (n = 37; 51.4%) and *S.* Typhi (n = 20; 27.8%) followed by *S.* Enteritidis (n = 7; 9.7%), *S.* Typhimurium (n = 4; 5.6%), *S.* Paratyphi A (n = 2; 2.8%), *S.* London and *S.* Amsterdam (n = 1; 1.4% each).

The mean age of patients with *Salmonella* BSI was 34.2 years (range 8–71), 51.7% were women. They came from at least 10 different provinces, mainly the greater Phnom Penh area (n = 11; 19.0%) and Kandal province (n = 7; 12.1%). The majority of *Salmonella* BSI occurred during the rainy months April to November (n = 57; 79.1%); no apparent other temporal or geographical clustering was noted.

Co-morbidity was present in 36 (62.1%) patients, mainly human immunodeficiency virus (HIV) infection (n = 32; 55.2%); we also noted systemic lupus erythematodes (n = 2; 3.4%), thalassemia and valvular heart disease (one patient each). For 13 HIV-infected patients, *Salmonella* BSI was the indicator disease for HIV-infection; only three HIV-patients were on antiretroviral treatment at the time of the BSI. The median CD4-cell count was 22 per microliter (range 2–253), concurrent opportunistic infections (OI) included tuberculosis (n = 6) and cryptococcal meningitis (n = 3). Of note, *S.* Choleraesuis was the most common pathogen in HIV-infected patients (21/32, 65.6%) whereas *S.* Typhi was predominantly recovered from HIV-negative patients (19/26, 73.1%). Of the 24 patients with *S.* Choleraesuis BSI, 12 (50.0%) presented with fever, six (25.0%) with abdominal pain and diarrhea and five (20.8%) with dyspnea and dry cough.

Patients were treated empirically with either ceftriaxone, amoxicillin-clavulanic acid or ciprofloxacin (or subsequent administration of these antibiotics) for a mean duration of 11.7 days (range 1–21). Additional treatment for HIV-related OI included SMX-TMP, fluconazole and tuberculostatic drugs.

A total of five patients infected with *Salmonella* spp. (8.6%) had one or more recurrent BSI episodes with the same serovar, all *S.* Choleraesuis (Figure 1). The mean interval to recurrence was 4.5 weeks (range 2–10 weeks). One HIV-patient had a *S.* Typhimurium BSI eight months after being treated for *S.* Enteritidis BSI.

Five patients (8.6%) died. Four of them had been infected by *S.* Choleraesuis and one by *S.* Typhimurium. All were HIV-infected with advanced immune depression; at least three of them suffered from concurrent life-threatening opportunistic infections (tuberculosis n = 2, cryptococcal meningitis n = 1). The median duration between the diagnosis of *Salmonella* BSI and death was 24 days (range 13–61 days)

### PFGE

For *S.* Choleraesuis, three different PFGE profiles were obtained, of which Xb-Chol-1 was predominant (86.5%), including all 13 recurrent isolates (data not shown). The PFGE profiles of ‘first’ and ‘recurrent’ isolates were identical per patient. No association between a particular PFGE profile and resistance profile was observed.

All *S.* Typhi isolates had a similar PFGE profile (*i.e.*Xb-Ty-1) whereas *S.* Enteritidis and *S.* Typhimurium presented with two and three different profiles respectively.

### Antibiotic resistance

Antibiotic resistance data as assessed for the 59 ‘first’ (*i.e*. non-recurrent) isolates are shown in Table 1. Of note, very high rates of MDR were seen in *S.* Typhi (15/20 isolates, 75.0%) and *S.* Choleraesuis (22/24 isolates, 91.7%) and to a lesser extent in other NTS (5/13 isolates, 38.5%).

**Table 1:** Antibiotic resistance in 59 Salmonella isolates (first BSI episode only), SHCH 2007–2011.

	resistant isolates			
	(%)			(n)
Antibiotic	*S.* Typhi (n = 20)	*S.* Choleraesuis (n = 24)	other NTS (n = 13)	*S.* Paratyphi A (n = 2)
Multi drug resistance^a^	75,0	91,7	38,5	0/2
Fluoroquinolone resistance				
Nalidixic acid	90,0	33,3	38,5	0/2
Decreased ciprofloxacin susceptibility (DCS)^b^	90,0	20,8	53,8	0/2
High level ciprofloxacin resistance^c^	0,0	0,0	7,7	0/2
Second line antibiotics				
Azithromycin^d^	5,0	70,8	15,4	0/2
Cefotaxim^e^	0,0	4,2	0,0	0/2
Combined resistance				
MDR+DCS	70,0	16,7	23,1	0/2
MDR+DCS+Azithromycin	0,0	4,2	7,7	0/2
Reserve antibiotics				
Meropenem	0,0	0,0	0,0	0/2
Tigecyclin	0,0	0,0	0,0	0/2
Fosfomycin	0,0	0,0	0,0	0/2

a *co-resistance to ampicillin+SMX-TMP+chloramphenicol*;

b *MIC ciprofloxacin >0.064 µg/ml, see text for details*;

c *MIC ciprofloxacin ≥4 µg/ml*;

d *MIC azithromycin >16 µg/ml*;

e *not included: 1 isolate S. Choleraesuis from recurrent infection, ESBL producing*.

DCS was particularly present among *S.* Typhi isolates, with MIC50 and MIC90 of 0.25 µg/mL and 0.38 µg/mL respectively (Table 2). Thirty-one (88.6%) out of 35 DCS isolates displayed resistance to NA, with mutations in *gyrA* at either position 83 (n = 24) or 87 (n = 3) (Table 3). One *S.* Typhimurium displayed full resistance to ciprofloxacin (MIC 6 µg/mL) confined to two mutations in *gyrA* (Ser83→Phe and Asp87→Asn) and one in *parC* (Ser80→Arg). Of note, four isolates (all NTS) displayed DCS but were NA susceptible: no mutations in *gyrA* or *parC* were observed; in two of them presence of *qnrS1* was detected. In 22 of 24 *S.* Choleraesuis and in all *S.* Paratyphi A we detected a *parC* mutation in position 57, regardless of susceptibility patterns.

**Table 2:** Distribution of minimal inhibitory concentration (MIC) for ciprofloxacin in 59 *Salmonella* isolates (first BSI episode only).

	MIC ciprofloxacin (µg/ml)^a^														
Serovar (n)	0.004	0.006	0.008	0.012	0.016	0.032	0.064	0.094	0.125	0.19	0.25	0.38	6	MIC 50	MIC 90
*S.* Choleraesuis (24)	2	5	5	2	2	-	-	3	4	1	-	.	-	0.012	0.125
*S.* Paratyphi A (2)	-	-	-	1	1	-	-	-	-	-	-	-	-	NA	NA
*S.* Typhi (20)	-	1	-	1	-	-	-	-	1	4	10	3	-	0.25	0.38
other NTS (13)	-	-	3	-	-	-	1	1	2	2	1	2	1	0.125	0.38

*NA, not applicable*.

a *resistance breakpoint 0.064 µg/ml*.

**Table 3:** Mutations in Gyrase and Topoisomerase and presence of qnr genes, according to serovar and resistance phenotype in 59 *Salmonella* spp.

Resistance phenotype	MIC ciprofloxacin	Serovars	n isolates	*gyrA*	*gyrB*	*parC*	qnr
Na^S^ Cip^S^ (n = 24)	0.004–0.064	*S.* Typhi	2	Glu133→Gly^b^ (n = 2)	-	-	-
		*S.* Paratyphi A	2	-	-	Thr57→Ser^c^ (n = 2)	-
		*S.* Choleraesuis	16	-	-	Thr57→Ser (n = 16)	-
		other NTS	4	Ile125→Ser^d^ (n = 1)	-	-	-
Na^S^ DCS (n = 4)	0.125–0.38	other NTS	4	-	-	Thr57→Ser (n = 1)	S1 (n = 2)
Na^R^ DCS (n = 31)	0.094–0.38	*S.* Typhi	18	Ser83→Phe/Glu133→Gly (n = 18)	-	-	-
		*S.* Choleraesuis^a^	8	Ser83→Phe (n = 2)	-	Thr57→Ser (n = 7)	-
				Ser83→Tyr (n = 2)	-	-	-
				Asp87→Gly (n = 1)	-	-	-
				Asp87→Tyr (n = 1)	-	-	-
		other NTS	4	Ser83→Ile (n = 2)	-	-	S1 (n = 1)
				Asp87→Tyr (n = 1)	-	-	-
Na^R^ Cip^R^ (n = 1)	6	*S.* Typhimurium	1	Ser83→Phe/Asp87→Asn	-	Ser80→Arg	-

*NaS, nalidixic acid susceptible; CipS, ciprofloxacin susceptible; NaR, nalidixic resistant; DCS, decreased ciprofloxacin susceptibility*.

a *co-presence of Ser 83(gyrA) and Thr57 (parC) mutations in 4 isolates*;

b *Glu133→Gly: silent mutation*;

c *Thr57→Ser: silent mutation;*

d *Ile125→Ser: silent mutation*.

MIC levels for azithromycin were particularly high in *S.* Choleraesuis isolates, with MIC50 and MIC 90 as high as 32 and 128 µg/ml respectively (Table 4).

**Table 4:** Distribution of minimal inhibitory concentration (MIC) for azithromycin in 59 *Salmonella* isolates.

	MIC azithromycin (µg/ml)^a^																
Serovar (n)	1.5	2	3	4	6	8	12	16	24	32	48	64	96	128	>256	MIC 50	MIC 90
*S.* Choleraesuis (24)	1	1	3	2	-	-	-	-	-	6	1	3	2	3	2	32	128
*S.* Paratyphi A (2)	-	-	-	1	1	-	-	-		-	-	-	-	-	-	NA**	NA
*S.* Typhi (20)	-	1	6	10	2	-	-	-	-	-	-	-	1	-	-	4	6
other NTS (13)	1	2	3	4	1	-	1	-	-	-	-	1	-	-	-	4	12

*NA, not applicable*.

a *epidemiological cutoff point 16 µg/ml*.

In the successive isolates from patients with recurrent *Salmonella* BSI, no differences in resistance patterns were noted, except in one *S.* Choleraesuis (recovered 23 days after the first *S.* Choleraesuis BSI episode), having acquired ESBL. Presence of ESBL was also detected in another patient with *S.* Choleraesuis infection. Both ESBL-positive isolates carried *bla*_CTX-M_ genes, The former was confirmed as CTX-M group 9 and displayed also MDR and azithromycin resistance (MIC 32 µg/mL). In the latter (CTX-M group 1), we observed additional DCS (MIC 0.125 µg/mL).

## Discussion

We described the serovar distribution and antibiotic susceptibility of 72 *Salmonella enterica* BSI isolates from Cambodian adults, and noted a predominance of *S.* Typhi and *S.* Choleraesuis. Besides MDR, *S.* Typhi in particular displayed high rates of DCS, while *S.* Choleraesuis was associated with advanced HIV-infection and remarkably high azithromycin resistance rates.

Our findings have several limitations. The study describes *Salmonella* BSI mainly in adults. As *Salmonella* spp. is an important pediatric pathogen in tropical low-resource settings [1], [3], data on its invasive infections in children are essential to complement the epidemiological picture of salmonellosis in Cambodia. Next, our clinical hospital data did not allow calculations of incidence and/or the true burden of disease because the population denominator and referral pattern were not known. In addition, the presence of an HIV-treatment center in the hospital may have led to a patient selection bias. In spite of these limitations our data shed new light on invasive *Salmonella* infections in Cambodia.

In HIV-negative patients, *S.* Typhi was the most common serovar, with very high rates of MDR (75.0%) and DCS (90.0%). This confirms earlier trends from Cambodia as noted by Kasper and coworkers in 2009 [21] describing 56% of MDR and 80% DCS in *S.* Typhi. The presence of MDR and DCS has been observed in other Asian countries, albeit with important differences. A survey on typhoid fever in five countries [22] revealed MDR rates as variable as 65% in Pakistan, 22% in Vietnam, 7% in India and 0% in China/Indonesia whereas rates of NA resistant S. Typhi (NARST) ranged similarly between 57–59% (India, Pakistan), 44% (Vietnam) and 0% (China, Indonesia). Since the early 1990's, Southern Vietnam has been particularly mentioned as a regional ‘typhoid resistance hotspot’ with NARST/DCS rates as high as 90–98% [10], [23]. The geographical location of Cambodia in the vicinity of this regional ‘hotspot’ may be one of the explanations for the high rates of DCS among our patients with typhoid fever, given the intense cross-border traffic between the two countries. In addition, the uncontrolled use of ciprofloxacin and other antibiotics and the limited access to safe water and sanitation services [24] probably add to selection and spread of MDR and DCS isolates.

In Vietnam, the Ser83→Phe substitution in *gyrA* was described as the predominant underlying resistance mechanism for DCS [23]. We observed this mutation also in all *S.* Typhi isolates with combined DCS and NA resistance and to a lesser extent in *S.* Choleraesuis and other NTS. According to the Cambodian National Treatment Guidelines [25] ciprofloxacin is the first choice treatment for presumed typhoid fever with ceftriaxone as alternative. Given the failure risk of a treatment course with ciprofloxacin for invasive salmonellosis with DCS as high as 36% [26], we think the empiric treatment of typhoid fever with ciprofloxacin should be abandoned in Cambodia. Alternatives could be azithromycin for uncomplicated cases and ceftriaxone for hospitalized patients. Gatifloxacin proved to be a safe, cheap and effective alternative treatment in Nepal [27] and Vietnam [28], but it is not widely distributed in Cambodia, and caution remains regarding its use in the elderly and in a setting with increasing rates of MDR tuberculosis.

In addition, these data and their subsequent therapeutic challenges urge the need for more and better yet affordable diagnostic microbiology in Cambodia. More and adequately working microbiology laboratories across the country are essential for the improvement of clinical care and for surveillance of bacterial resistance.

Among HIV-infected patients, *S.* Choleraesuis was the most common serovar. It is a zoonotic pathogen causing paratyphoid in pigs and is an emerging cause of invasive infections in immune compromised patients in Southeast and Eastern Asia [29]. The prevalence of *S.* Choleraesuis was not yet described in Cambodia in swine nor in humans but it is a well-known pathogen in neighboring Thailand [30], [31].

All isolates in patients with recurrent *S.* Choleraesuis BSI had PFGE profiles which were identical to the first isolate, which is suggestive for relapse rather than for reinfection although the small number of pulsotypes and the limited discriminatory power of PFGE using XbaI [32] should be taken into account. Given the context of advanced HIV-infection, relapse is the more likely interpretation [33]

Most *S.* Choleraesuis isolates (70.8%) had azithromycin MIC-values exceeding 16 µg/mL. To our knowledge, this has not yet been described in a series of clinical *Salmonella* isolates from a single setting. Of note, also one *S.* Typhi and *S.* Enteritidis isolate displayed high azithromycin MIC-values. This contrasts with the low azithromycin MIC data for *S.* Typhi reported from Vietnam (MIC90 8–16 µg/mL [10], [34]), India and Egypt (MIC90 8 µg/mL [35], [36]). Azithromycin MIC-values up to 64 µg/mL in *S.* Typhi and Paratyphi A from India were recently described [37], and a Finnish study revealed azithromycin MIC-values ≥32 µg/mL in 1.9% of 1237 NTS isolates; half of them were isolated after travel to Thailand [38]. While considering the azithromycin resistance ‘epidemiological cutoff’ of 16 µg/mL [17], azithromycin resistance apparently presents an emerging problem as treatment failures have been described [39].

Possible mechanisms of azithromycin resistance include the presence of specific resistance genes (e.g. *mph*A, *mph*B, *erm*B), a mutation in *rlp*D or *rlp*V, or the acquisition of an efflux pump [40]. In Cambodia, generic azithromycin can be purchased over the counter of private clinics and pharmacies; local prices vary between 1 to 5 US $ per tablet. It is commonly used for respiratory tract infections, and often prescribed when all other treatments have failed (personal communication Thong Phe). No local data about macrolide use in animals are available, but a recent report from Vietnam showed that antibiotics such as macrolides, lincomycin, colistin, and aminoglycosides are actually used in livestock [41].

As the above mentioned azithromycin resistance in our study is most prevalent in *S.* Choleraesuis, our findings may firstly affect empiric treatment choices for fever and presumed BSI in HIV-infected patients. Given the complex resistance patterns in *S.* Choleraesuis, neither ciprofloxacin nor azithromycin appear to be safe choices; the most likely alternative in the Cambodian setting is probably a third generation cephalosporin. However, in two *S.* Choleraesuis isolates the presence of ESBL was found. Extensive antibiotic resistance, including ESBL has been reported before for *S.* Choleraesuis in East Asia [42], [43]. Even though ESBL prevalence in *Salmonella enterica* is still low compared to the very high rates in community-acquired *Escherichia coli* and *Klebsiella pneumoniae* isolates in the same study population [44], this is a very worrisome trend, as the potential for transmission of resistance genes is expected.

These results warrant further surveillance of resistance in invasive bacterial pathogens and *Salmonella* spp. in particular in Cambodia. More in depth research of the causes and molecular mechanisms of this *in vitro* measured azithromycin resistance are needed. In addition, integrated research on the human and veterinary epidemiology of *S.* Choleraesuis in Cambodia is essential for better understanding of the disease dynamics and planning of public health interventions.

## Conclusions

*S.* Typhi and *S.* Choleraesuis are both common *Salmonella* serovars causing BSI in Cambodian adults; *S.* Choleraesuis closely associated with advanced HIV-disease. DCS and azithromycin resistance are very high in *S.* Typhi and *S.* Choleraesuis respectively, while presence of ESBL is emerging. Human salmonellosis has become a difficult-to-treat infection in Cambodia requiring close surveillance and public health attention.

## Supporting Information

Checklist S1 STROBE checklist.
(DOC)
